# Omicron XBB.1.16-Adapted Vaccine for COVID-19: Interim Immunogenicity and Safety Clinical Trial Results

**DOI:** 10.3390/vaccines12080840

**Published:** 2024-07-25

**Authors:** María Jesús López Fernández, Silvia Narejos, Antoni Castro, José María Echave-Sustaeta, María José Forner, Eunate Arana-Arri, José Molto, Laia Bernad, Raúl Pérez-Caballero, Julia G. Prado, Dàlia Raïch-Regué, Rytis Boreika, Nuria Izquierdo-Useros, Benjamin Trinité, Julià Blanco, Joan Puig-Barberà, Silvina Natalini Martínez

**Affiliations:** 1 Servicio de Medicina Preventiva y Salud Pública, Hospital Regional Universitario de Málaga, 29010 Málaga, Spain; 2 Centro de Atención Primaria Centelles, 08540 Centelles, Spain; 3 Hospital Universitari de Girona Doctor Josep Trueta, 17007 Girona, Spain; 4 Hospital Universitario Quirónsalud Madrid, 28223 Madrid, Spain; 5 Hospital Clínico Universitario Valencia, 46010 Valencia, Spain; 6 Unidad de Coordinación Científica, Biocruces Bizkaia, Osakidetza, 48903 Barakaldo, Spain; 7 Centro de Investigación Biomédica en Red-Enfermedades Infecciosas (CIBERINFEC), Instituto de Salud Carlos III, 28029 Madrid, Spain; 8 Department of Infectious Diseases, Fundació Lluita Contra les Infeccions, Hospital Universitari Germans Trias i Pujol, 08916 Badalona, Spain; 9 IrsiCaixa, Can Ruti Campus, 08916 Badalona, Spainrperez@irsicaixa.es (R.P.-C.);; 10 Institut de Recerca Germans Trias i Pujol (IGTP), 08916 Badalona, Spain; 11 Càtedra de Malalties Infeccioses i Immunitat, Facultat de Medicina, Universitat de Vic-Universitat Central de Catalunya (UVic-UCC), 08500 Vic, Spain; 12 Área de Investigación en Vacunas, Fundació per al Foment de la Investigació Sanitària i Biomèdica de la Comunitat Valenciana (FISABIO), 46020 Valencia, Spain; jpuigb55@gmail.com; 13 Unidad de Investigación de Vacunas, Instituto de Investigación Sanitaria HM, 28938 Madrid, Spain

**Keywords:** JN.1, XBB.1.16, adapted vaccine, SARS-CoV-2 vaccine, adjuvanted protein vaccine, booster vaccine, COVID-19

## Abstract

(1) Background: The global coronavirus disease 2019 vaccination adapts to protect populations from emerging variants. This communication presents interim findings from the new Omicron XBB.1.16-adapted PHH-1V81 protein-based vaccine compared to an XBB.1.5-adapted mRNA vaccine against various acute respiratory syndrome coronavirus 2 (SARS-CoV-2) strains. (2) Methods: In a Phase IIb/III pivotal trial, adults previously vaccinated with a primary scheme and at least one booster dose of an EU-approved mRNA vaccine randomly received either the PHH-1V81 or BNT162b2 XBB.1.5 vaccine booster as a single dose. The primary efficacy endpoint assessed neutralization titers against the Omicron XBB.1.16 variant at day 14. Secondary endpoints evaluated neutralization titers and cellular immunity against different variants. Safety endpoints comprised solicited reactions up to day 7 post-vaccination and serious adverse events until the cut-off date of the interim analysis. Changes in humoral responses were assessed by pseudovirion-based or virus neutralization assays. (3) Results: At the cut-off date, immunogenicity assessments included 599 participants. Both boosters elicited neutralizing antibodies against XBB.1.16, XBB.1.5, and JN.1, with PHH-1V81 inducing a higher response for all variants. The PHH-1V8 booster triggers a superior neutralizing antibody response against XBB variants compared to the mRNA vaccine. A subgroup analysis consistently revealed higher neutralizing antibody responses with PHH-1V81 across age groups, SARS-CoV-2 infection history, and the number of prior vaccination shots. A safety analysis (n = 607) at the day 14 visit revealed favorable safety profiles without any serious vaccine-related adverse events. (4) Conclusions: PHH-1V81 demonstrates superiority on humoral immunogenicity compared to the mRNA vaccine against XBB variants and non-inferiority against JN.1 with a favorable safety profile and lower reactogenicity, confirming its potential as a vaccine candidate.

## 1. Introduction

The coronavirus disease 2019 (COVID-19) has spurred extensive vaccination efforts worldwide to combat the severe acute respiratory syndrome coronavirus 2 (SARS-CoV-2) and its variants [1,2,3]. While current vaccines have shown effectiveness, their protection diminishes over time, particularly against emerging variants [4,5,6]. This highlights the need to adapt vaccine compositions and develop updated strategies to sustain immunity [7].

In response to Omicron XBB variants, global health authorities, including the World Health Organization Technical Advisory Group on COVID-19 Vaccine Composition, the European Centre for Disease Prevention and Control, and the European Medicines Agency, recommended updates to vaccine formulations [8,9]. mRNA and protein-based vaccines targeting Omicron XBB have gained approval, addressing evolving variants effectively [9,10,11,12,13]. The emergence of JN.1 highlights the ongoing pandemic challenges, emphasizing the critical need for vaccine advancement [14,15].

PHH-1V, a bivalent protein-based adjuvanted vaccine, has emerged as a promising booster [16]. Phase IIb trials demonstrated its efficacy in generating neutralizing antibodies against SARS-CoV-2 variants, including Omicron XBB [16,17,18]. A long-term analysis revealed sustained immune responses, even in high-risk populations regardless of prior infection or primary vaccine type [19]. Additionally, PHH-1V induces a robust T-cell response [17,18].

Further, recipients of PHH-1V have reported fewer adverse events (AEs) than mRNA vaccine recipients, with comparable breakthrough non-severe COVID-19 rates. As a recombinant protein-based vaccine, PHH-1V offers advantages such as high productivity, stability, and suitability for immunocompromised individuals [20]. PHH-1V81, a newly adapted XBB vaccine, targets XBB.1.16 receptor-binding domain (RBD) homodimer and was developed in response to the evolution of SARS-CoV-2. Like PHH-1V, PHH-1V81 is a recombinant protein-based vaccine but is tailored to enhance protection against the more recent Omicron variants [16].

Here, interim findings from the HIPRA-HH-14 clinical study are reported. The HIPRA-HH-14 is a Phase IIb/III pivotal non-inferiority trial evaluating the immunogenicity and safety of the XBB.1.16 monovalent-adapted vaccine, PHH-1V81, as a booster. It examines immunogenicity changes against various SARS-CoV-2 variants, including Omicron XBB.1.16, Omicron XBB.1.5, and emerging variants such as JN.1, in a subset of participants. 

## 2. Materials and Methods

### 2.1. Study Design

This HIPRA- HH-14 Phase IIb/III pivotal trial was a double-blind, randomized, active-controlled, multi-center, non-inferiority clinical study assessing the immunogenicity, safety, and tolerability of PHH-1V81 XBB.1.16-adapted booster vaccine, targeting the Omicron XBB.1.16 variant of SARS-CoV-2 compared to an mRNA XBB.1.5-adapted vaccine. Participants previously received two doses of an EU-approved mRNA vaccine and at least one booster dose.

The PHH-1V (BIMERVAX^®^; HIPRA) antigen is a heterodimer based on the fusion of two RBDs from SARS-CoV-2 spike protein, incorporating the RBDs from the B.1.351 (Beta) and B.1.1.7 (Alpha) variants into a single peptide using recombinant deoxyribonucleic acid (DNA) technology. Expressed in a Chinese Hamster Ovary cell line, each 0.5 mL dose contains 40 µg of the active substance in phosphate-buffered saline with a squalene (SQBA) adjuvant [16]. The PHH-1V81 antigen is a homodimer of two consecutive copies of the RDB from the Omicron XBB.1.16 variant and is being developed as a booster dose, focusing on providing robust immunity against the prevalent Omicron sublineage [5,16].

Participants in the experimental arm received a single intramuscular dose of 40 µg of PHH-1V81, while subjects in the active comparator arm received a single intramuscular dose of 30 µg of Comirnaty Omicron XBB1.5 [21]. 

The primary efficacy endpoint is neutralization titers against the Omicron XBB.1.16 variant at day 14. Secondary efficacy endpoints include neutralization titers against the Omicron XBB.1.16 variant at days 91 and 182 and against the Wuhan, Omicron BA.1, Omicron XBB.1.5 strains at days 14, 91, and 182. Safety endpoints comprise solicited systemic and local reactions up to day 7 post-vaccination, unsolicited AEs up to day 28 after vaccination, AEs of special interest (AESI) until study end, medically attended AEs, and serious AEs (SAE) throughout the study. 

The interim analysis compares the immunogenicity and safety of PHH-1V81 (HIPRA) with BNT162b2 XBB.1.5 (Pfizer [New York, NY, USA], BioNTech, Mainz, Germany) at baseline and day 14, including solicited AE at day 7 post-vaccination for participants who completed the day 14 visit, and SAE until the interim analysis cutoff date (12 December 2023). 

Humoral response was evaluated by measuring the inhibitory concentration 50 (IC50) using a pseudovirion-based neutralization assay (PBNA) [22,23,24] against Omicron XBB.1.16 and Omicron XBB.1.5 variants, reported as geometric mean titer (GMT) and geometric mean fold rises (GMFR) for adjusted treatment.

Cellular responses were assessed through interferon-γ (IFN-γ) ELISpot assay as an exploratory endpoint. With this purpose, peripheral blood mononuclear cells (PBMCs) were re-stimulated in vitro with 6 SARS-CoV-2 RBD peptides’ pools (Wuhan, China, Omicron BA.1, Omicron XBB.1.5, Omicron XBB.1.16, Omicron BA.2.86, and Omicron JN.1 variants) at baseline and day 14 in a subset of participants to determine the percentage of antigen-specific IFN-γ-producing T-cells [18].

In addition to planned immunogenicity assessments, a virus neutralization assay (VNA) was conducted in a random subset of serum samples to compare humoral immune response between vaccine arms against the Omicron JN.1 [25,26].

### 2.2. Participants

The trial enrolled adults aged 18 or older who provided informed consent, received a primary series of two doses and at least one booster dose of an EU-approved mRNA vaccine with last dose at least six months before inclusion, and tested negative for acute SARS-CoV-2 infection on day 0. Eligible participants had stable chronic diseases, while those with a previous SARS-CoV-2 infection must have been diagnosed at least 6 months before day 0 (Table S1). The trial, conducted at ten clinical sites in Spain, began enrollment on 15 November 2023 and, due to rapid recruitment, closed on 29 November 2023.

### 2.3. Randomization and Treatment Allocation

This double-blinded study ensured that participants, site staff (including those involved in drug preparation and administration), laboratory analysts, the sponsor, and the Clinical Research Organization (CRO) were unaware of the treatment allocations. Only unblinded hospital pharmacists or other qualified personnel prepared the booster doses, and unblinded staff members, who were not otherwise involved in the study procedures except for blood extraction, administered the treatments [18]. Blinding was maintained until study finalization. A label system was used to disguise the syringe contents due to the visual distinction between the two vaccines.

Participants were randomly assigned to treatment arms in a 2:1 ratio to receive either a booster dose of PHH-1V81 (HIPRA XBB.1.16-adapted vaccine, n = 408) or a booster dose of BNT162b2 Omicron XBB.1.5 (Comirnaty^®^ Omicron XBB.1.5, Pfizer-BioNTech adapted vaccine, n = 204) using Interactive Response Technology (IRT). The allocation was stratified by age group, with approximately 90% of participants aged 18–64 years and 10% aged 65 years or older [18]. An independent statistician, who is not involved in the study, generated the randomization scheme. Upon enrollment, each participant was assigned a randomization number exclusively for arm allocation.

### 2.4. Sample Size

In the HIPRA-HH-14 trial, sample size determination aimed to confirm PHH-1V81’s non-inferiority to the comparator vaccine in inducing neutralizing antibody titers against Omicron XBB variants. For consistency with previous HIPRA-HH studies, the success criterion was defined as the upper bound of the two-sided 95% confidence interval (CI) around the GMT ratio BNT162b2 XBB.1.5/PHH-1V81, which should lie below 1.50. Superiority is demonstrated if the upper bound of the 95% CI of the GMT ratio BNT162b2: PHH-1V81 is below 1 [27]. With a 2:1 randomization ratio, group sizes of 366 and 183 ensured 90% power at a one-sided 2.5% significance level. This resulted in 612 randomized subjects, with 408 in the PHH-1V81 arm.

### 2.5. Statistical Analysis

Descriptive analyses compared time points and treatment arms for each SARS-CoV-2 variant. Categorical variables were presented as cases and percentages, while continuous variables included non-missing observations, mean (or geometric mean), standard deviation (or geometric standard deviation), median, interquartile range, minimum, and maximum, without imputation for missing data. Efficacy analyses followed predefined hypotheses for non-inferiority, with the upper bound of the 95% CI determining claim validation. GMT and GMFR adjusted treatment for immunogenicity endpoints were estimated using Mixed Models for Repeated Measures (MMRM), while T-cell data were analyzed with mixed effects models. Values below the lower limit of quantification (LLOQ) were imputed as LLOQ, and PBNA values exceeding 20,480 were reanalyzed. 

### 2.6. Ethical Considerations

The trial was conducted in accordance with national and international regulations [28,29,30]. The study protocol was approved by the Spanish Agency of Medicines and Medical Devices (AEMPS) and by HM Hospitals Research Ethics Committee (23.10.2249-GHM). Trial registration numbers are NCT06181292; EU CT No: 2023-508458-25-00.

## 3. Results

**Figure 1:**
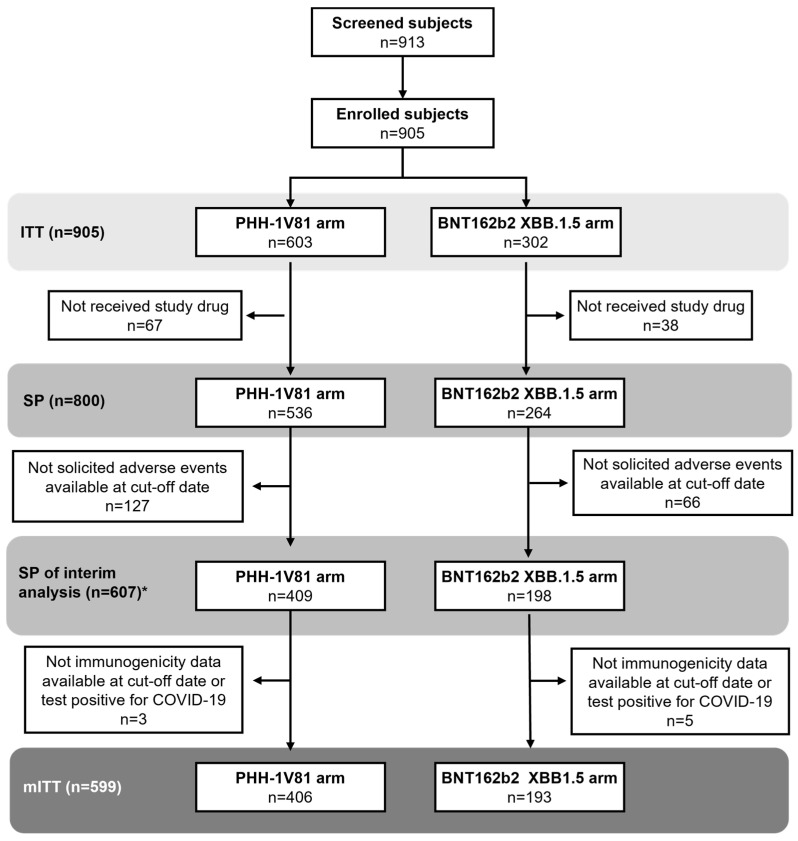
The participants’ disposition of HIPRA-HH14. The Enrolled Population (EP) is defined as all subjects who signed the Informed Consent Form. The Intention-to-treat Population (ITT) is defined as all subjects of the EP who are randomly assigned to treatment, regardless of the subject’s treatment status in the study. The safety population (SP) set is defined as all randomized subjects who received the study drug. A total of *607 subjects (409 in PHH-1V81 arm and 198 in the BNT162b2 XBB.1.5 arm) had completed the day 14 visit, and information on solicited adverse events was available at the cut-off date set on 12 December 2023. The modified ITT Population (mITT) is defined as all participants in the ITT who met the inclusion/exclusion criteria and received a dose of study drug, whose baseline and day 14 were available and who did not test positive for COVID-19 within 14 days of receiving the study drug. COVID-19: coronavirus disease.

Out of 913 screened, 905 subjects constituted the Intention-to-Treat (ITT) population (293 more than the calculated sample size). Among them, 603 were in the PHH-1V81 arm and 302 in the BNT162b2 XBB.1.5 arm. Vaccine administration was confirmed for 800 subjects (536 in PHH-1V81; 264 in BNT162b2 XBB.1.5) as of 12 December 2023, forming the safety population for the interim analysis. Only 607 subjects (409 in PHH-1V81; 198 in BNT162b2 XBB.1.5) completed the day 14 visit with solicited AE information available for the interim analysis. Immunogenicity data at both baseline and day 14 visits were available for 599 participants (66.2% of ITT, 97.9% of target sample size), included in the modified Intention-to-Treat (mITT) population for immunogenicity assessments. Among them, 406 received the PHH-1V81, and 193 received the BNT162b2 XBB.1.5. No premature discontinuations occurred by the cutoff date (Figure 1).

### 3.1. Baseline Characteristics

Participants had a median age of 45 years (range: 18 to 88 years), with similar age distributions in both vaccine arms. Most subjects were female (59.3%), having received either three (66.9%) or four (33.0%) previous vaccination doses. Demographic characteristics were generally balanced between the vaccine arms (Table 1).

### 3.2. PHH-1V81 Immunogenicity against Omicron XBB.1.16 and Omicron XBB.1.5 Variants

**Figure 2:**
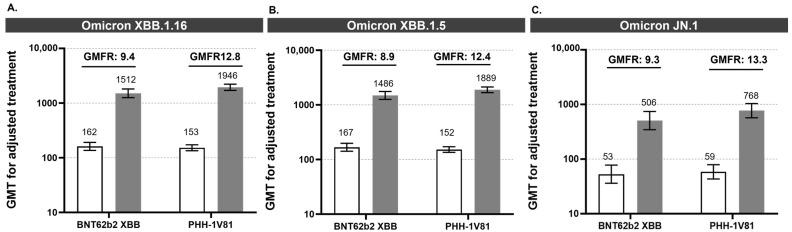
The humoral response against SARS-CoV-2 variants Omicron XBB.1.16, Omicron XBB.1.5, and Omicron JN.1 induced by BNT62n2 XBB and PHH-1V81 at day 14. (**A**) The GMT for the adjusted treatment against the Omicron XBB.1.16 variant for each vaccinated group (PHH-1V81 group; n = 406 and BNT62b2 XBB; n = 193) by PBNA; (**B**) the GMT for the adjusted treatment against the Omicron XBB.1.5 variant for each vaccinated group (PHH-1V81 group; n = 406 and BNT62b2 XBB; n = 193) by PBNA; (**C**) the GMT for the adjusted treatment against the Omicron JN.1 variant for a subset of n = 100 participants (n = 65 with PHH-1V81 and n = 35 with BNT62b2 XBB1.5) by VNA. Graphics (**A**–**C**) represent the mean GMT with 95% CI at baseline (with a bar) and 14 days after booster (dark grey bar); the upper numbers represent the mean GMFR at day 14 from the baseline. CI: confidence interval; GMT: geometric mean titer; GMFR: geometric mean fold rise.

Both vaccines show a significant increase in neutralizing antibodies 14 days post-booster immunization compared to baseline against the Omicron XBB.1.16 and Omicron XBB.1.5 variants (Figure 2 and Table S2). For the adjusted treatment, the GMT (95% CI) against Omicron XBB.1.16 increased from baseline 152.46 (134.72, 172.54) to 1946.38 (1708.44, 2217.46) at day 14 after the PHH-1V81 booster and from baseline 161.57 (136.40, 191.37) to 1512.21 (1261.72, 1812.44) at day 14 after the BNT162b2 XBB.1.5 booster. The GMT against Omicron XBB.1.5 increased from 151.93 (134.89, 171.13) to 1888.89 (1676.99, 2127.57) at day 14 after the PHH-1V81 booster and from 167.89 (142.04, 198.44) to 1486.03 (1257.25, 1756.45) at day 14 after the BNT162b2 XBB.1.5 booster. 

These increases in neutralizing antibodies are reflected in a GMFR (95% CI) for adjusted treatment at day 14 post-booster vaccination: 12.76 (11.01, 14.78) for PHH-1V81 and 9.42 (7.61, 11.66) for BNT162b2 XBB.1.5 against Omicron XBB.1.16; and 12.42 (10.62, 14.51) for PHH-1V81 and 8.88 (7.20, 10.94) for BNT162b2 XBB.1.5 against Omicron XBB.1.5.

**Figure 3:**
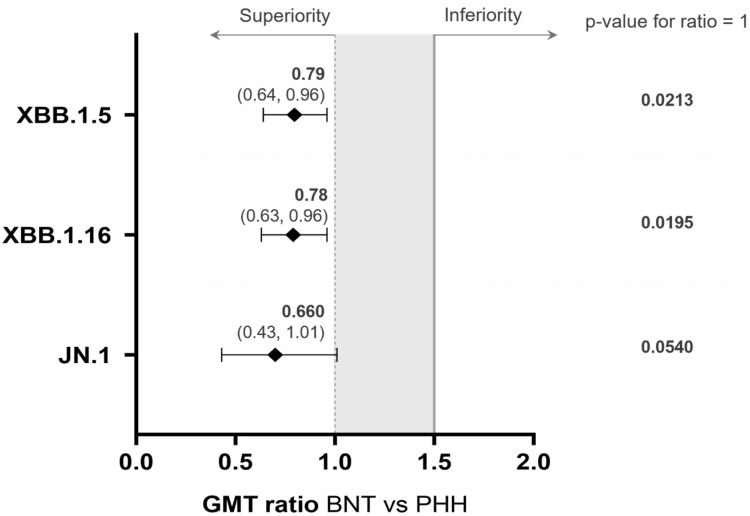
A comparison of the humoral responses elicited by BNT62b2 XBB and PHH-1V81 at day 14 against SARS-CoV-2 variants Omicron XBB.1.16, Omicron XBB.1.5, and Omicron JN.1. Forest plot for GMT ratio (95% CI) BNT62b2 XBB.1.5 vs. PHH-1V81 at day 14. The solid line indicates the non-inferiority limit of the trial (NIm = 1.5), and the dashed line indicates the superiority limit (GMT ratio = 1.0) [27]. BNT: BNT62b2 XBB1.5 vaccine; CI: confidence interval; GMT: geometric mean titer; GMFR: geometric mean fold rise; PHH: PHH-1V81 vaccine.

Comparing vaccine arms, higher neutralizing antibody levels were observed after boosting with PHH-1V81 compared to BNT162b2 XBB.1.5 against both analyzed SARS-CoV-2 variants, with significant differences in GMT at day 14 post-booster. The GMT ratio between vaccine arms at day 14 was 0.78 (95% CI: 0.63, 0.96; *p* < 0.05) against Omicron XBB.1.16 and 0.79 (95% CI: 0.64, 0.96; *p* < 0.05) against Omicron XBB.1.5, with no differences found at baseline (Figure 3). Additionally, significant differences in GMFR at day 14 were observed between vaccination arms (Table S2).

In the PHH-1V81 arm, 77.6% (95% CI: 73.2, 81.6%) and 76.4% (71.91, 80.41%) of subjects showed a ≥4-fold rise in neutralizing antibody titers against Omicron XBB.1.16 and Omicron XBB.1.5, respectively. In contrast, in the BNT162b2 XBB.1.5 arm, 71.0% (64.0, 77.3%) and 70.5% (63.49, 76.80%) of subjects showed a ≥4-fold rise in neutralizing antibody titers against the respective variants.

### 3.3. PHH-1V81 Immunogenicity against Omicron XBB.1.16 and Omicron XBB.1.5 Variants by Participant Subgroups

**Figure 4:**
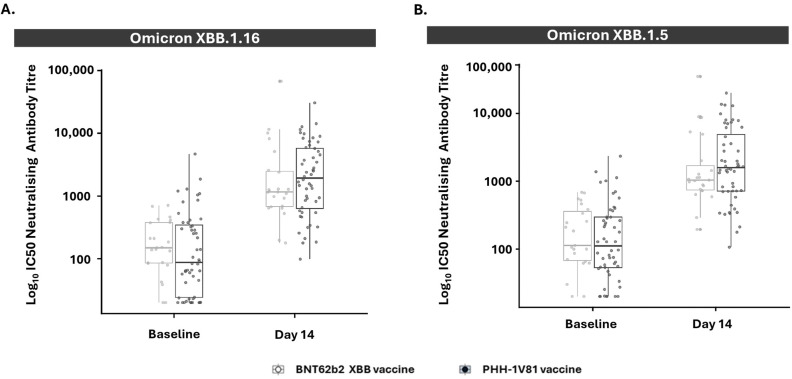
Neutralizing antibody responses against Omicron XBB.1.16 and Omicron XBB.1.5 variants in persons ≥60 years old at baseline and day 14 by vaccine arm (mITT population). (**A**) Neutralizing antibody titer against the Omicron XBB.1.16 variant on participants ≥ 60 years old (PHH-1V81, n = 52; BNT62b2 XBB, n = 23) for each vaccinated group; (**B**) neutralizing antibody titer against Omicron XBB.1.5 variant on participants ≥60 years old (PHH-1V81, n = 52; BNT62b2 XBB, n = 23) for each vaccinated group. Graphics represent individual log_10_ IC50 (dots) and box with median, IQR, and whiskers of 1.5 times IQR. IC50: half maximal inhibitory concentration; IQR: interquartile range; SARS-CoV-2: severe acute respiratory syndrome coronavirus 2.

Subgroup analyses on neutralizing antibody titers against the Omicron XBB.1.16 and Omicron XBB.1.5 at day 14 revealed that among participants aged 60 and above, PHH-1V81 generated higher neutralizing antibody levels against both variants compared to BNT162b2 XBB.1.5 (Figure 4 and Table S3).

**Figure 5:**
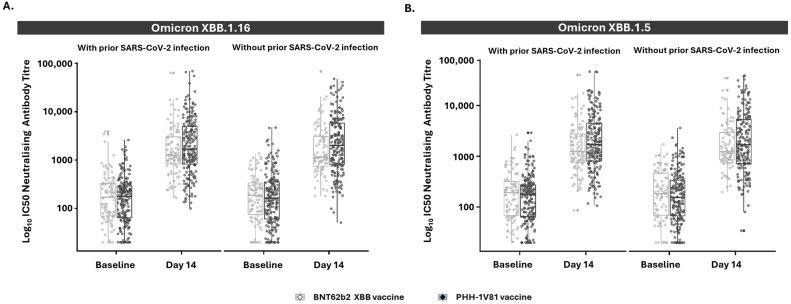
Neutralizing antibody responses against Omicron XBB.1.16 and Omicron XBB.1.5 variants in persons with and without prior reported SARS-CoV-2 infections at baseline and day 14 by vaccine arm (mITT population). (**A**) Neutralizing antibody titer against Omicron XBB.1.16 variant on participants with (PHH-1V81, n = 206; BNT62b2 XBB, n = 99) or without (PHH-1V81, n = 200; BNT62b2 XBB, n = 94) previous reported SARS-CoV-2 infection for each vaccinated group; (**B**) neutralizing antibody titer against Omicron XBB.1.5 variant on participants with (PHH-1V81, n = 206; BNT62b2 XBB, n = 99) or without (PHH-1V81, n = 200; BNT62b2 XBB, n = 94) previous reported SARS-CoV-2 infection for each vaccinated group. Graphics represent individual log_10_ IC50 (dots) and box with median, IQR, and whiskers of 1.5 times IQR. IC50: half maximal inhibitory concentration; IQR: interquartile range; SARS-CoV-2: severe acute respiratory syndrome coronavirus 2.

Similarly, individuals with and without prior SARS-CoV-2 infection exhibited increased neutralizing antibody levels following PHH-1V81 vaccination, surpassing levels induced by BNT162b2 XBB.1.5 (Figure 5 and Table S4).

**Figure 6:**
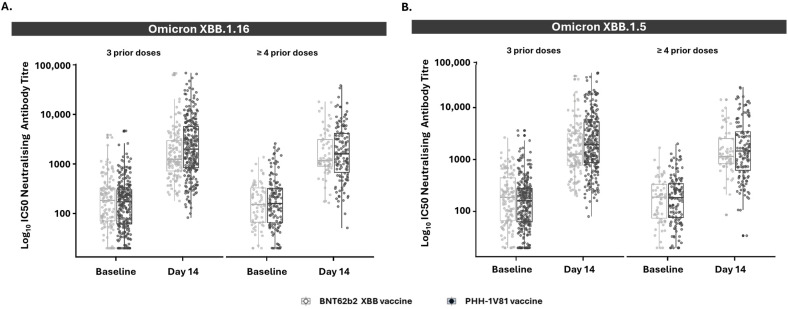
Neutralizing antibody responses against the Omicron XBB.1.16 and Omicron XBB.1.5 variants in persons with 3 or ≥4 prior doses of a COVID-19 vaccine at the baseline and day 14 by the vaccine arm (mITT population). (**A**) The neutralizing antibody titer against the Omicron XBB.1.16 variant on participants with 3 prior doses (PHH-1V81, n = 272; BNT62b2 XBB, n = 129) or ≥4 prior doses (PHH-1V81, n = 134; BNT62b2 XBB, n = 64) of a SARS-CoV-2 vaccine for each vaccinated group; (**B**) the neutralizing antibody titer against the Omicron XBB.1.5 variant on participants with 3 prior doses (PHH-1V81, n = 206; BNT62b2 XBB, n = 99) or ≥4 prior doses (PHH-1V81, n = 200; BNT62b2 XBB, n = 94) of a SARS-CoV-2 vaccine for each vaccinated group. Graphics represent individual log_10_ IC50 (dots) and box with median, IQR, and whiskers of 1.5 times IQR.IC50: half maximal inhibitory concentration; IQR: interquartile range.

Additionally, participants with three or more prior COVID-19 vaccine doses also showed numerically superior neutralizing antibody responses to Omicron XBB.1.16 and XBB.1.5 following PHH-1V81 boosting compared to BNT162b2 XBB.1.5 (Figure 6 and Table S5).

### 3.4. PHH-1V81 Immunogenicity against Omicron JN.1 Variant

The PHH-1V81 booster significantly increased neutralizing antibody titers against the Omicron JN.1 variant by VNA at day 14 post-booster compared to baseline titers for both vaccine arms. Analysis of neutralizing antibody titers against JN.1 involved a random subset of 100 participants (65 in PHH-1V81; 35 in BNT62b2). At day 14, PHH-1V81 showed numerically higher neutralizing antibodies against Omicron JN.1 compared to BNT62b2 Omicron XBB.1.5, with a GMT (95% CI) for adjusted treatment of 768.44 (568.96, 1037.86) and 505.88 (344.70, 742.43), respectively, and a GMT ratio of 0.66 (0.43, 1.01; *p* = 0.054), indicating non-inferiority of the PHH-1V81 booster against the JN.1 variant. The GMFR (95% CI) at day 14 was 13.34 (8.84, 20.12) for PHH-1V81 and 9.27 (5.70, 15.07) for BNT62b2 Omicron XBB.1.5 (Figure 2 and Figure 3 and Table S6).

### 3.5. PPH-1V81 Cellular Immunogenicity

SARS-CoV-2 specific T-cell responses post-booster were assessed in a subset of 40 participants (27 in PPH-1V81; 13 in BNT162b2 XBB.1.5) using ELISpot from PBMCs at baseline and day 14. The median age of participants was 44 years (range: 23 to 79), with four individuals aged 60 years or older (three in PHH-1V81; one in BNT162b2 XBB.1.5). 

**Figure 7:**
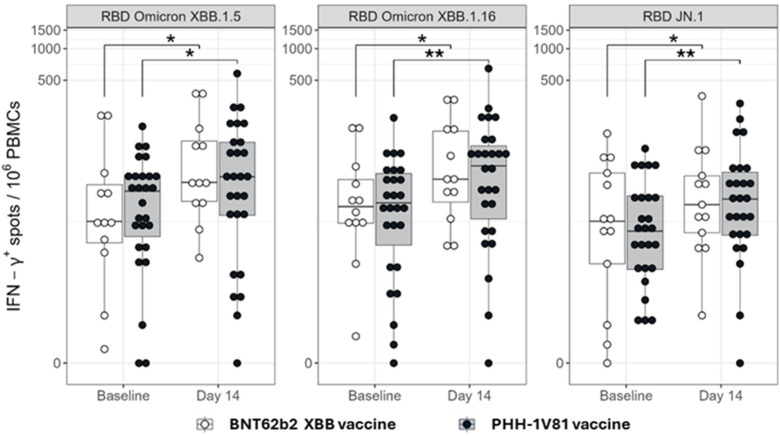
IFN-γ-producing T cells upon PBMC re-stimulation with SARS-CoV-2-derived peptide pools by the vaccine arm. The frequencies of IFN-γ responses determined by ELISpot assay in PBMCs from the subgroup of participants immunized with PHH-1V81 (n = 27) and BNT162b2 XBB.1.5 (n = 13). PBMCs were isolated before the boost immunization (baseline) and 2 weeks (D14) after the boost with PHH-1V81 and BNT162b2 XBB.1.5 vaccines; stimulated with RBD Omicron XBB.1.5, Omicron XBB.1.16, and Omicron JN.1 peptides’ pools; and analyzed by an IFN-γ-specific ELISpot assay. Within-group contrasts have been displayed in the plots, comparing the extent of IFN-γ+ response between timepoints in each treatment arm. Statistically significant differences between baseline and day 14 are shown in blue color as * *p* < 0.01; ** *p* < 0.001; IFN-γ: interferon-γ; PBMCs: peripheral blood mononuclear cells whole; RBD: receptor binding domain.

Both vaccines significantly increased the number of antigen-specific IFN-γ^+^ T-cells in response to in vitro PBMC re-stimulation with RBD peptide pools from the Omicron XBB.1.5, Omicron XBB.1.16, and Omicron JN.1 variants at day 14 post-booster compared to baseline. No significant differences were observed in IFN-γ^+^ spot forming cells between the vaccine arms (Figure 7 and Table S7).

### 3.6. Safety and Tolerability Results

Among the 607 participants who completed the day 14 visit and provided information on solicited AEs by 12 December 2023, 163 reported no AEs (118 (29%) in PHH-1V81; 45 (23%) in BNT162b2 XBB.1.5). 

**Figure 8:**
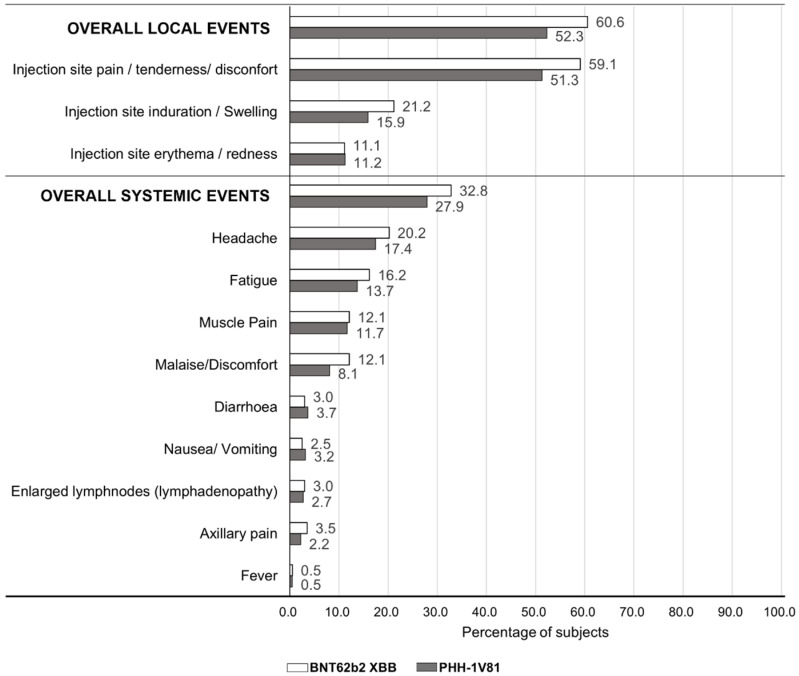
The percentage of subjects with solicited local and systemic adverse events through day 7 by vaccine arm. Solicited local adverse events and solicited systemic adverse events were reported MedDRA. PT from day 0 through day 7 for the safety population with the available data at the cut-off date. Data are shown as the percentage of subjects in relation to the safety population (n = 607; n = 409 in PHH-1V81 arm and n = 198 in BNT162b2 XBB.1.5 arm). If a subject experienced more than one event, the subject is counted once for each type of event. PTs are ordered in the decreasing frequency of the total number of subjects with each adverse event in the PHH-1V81 group. n, the number of subjects in the population; PT, preferred term.

Most solicited local AEs were mild, with 456 events in 210 subjects (51.3%) and 266 events in 118 subjects (59.6%) in the PHH-1V81 and BNT162b2 XBB.1.5 arms, respectively. Injection site pain, tenderness, and discomfort were the most commonly solicited local AEs, with a 59.1% and 51.3% incidence in the BNT162b2 XBB.1.5 and PHH1V81 arms, respectively (Figure 8). One subject (0.2%) in the PHH-1V81 arm reported a severe local solicited event. 

Regarding solicited systemic AEs, most were mild, with 369 events in 167 subjects (27.5%): 107 (26.2%) subjects in PHH-1V81 and 60 (30.3%) in BNT162b2 XBB.1.5. Severe systemic AEs were rare, affecting only four subjects (two (0.5%) in PHH-1V81; two (1.0%) in BNT162b2 XBB.1.5). The most frequent systemic AEs were headache, fatigue, and muscle pain, with higher incidences in the BNT162b2 XBB.1.5 arm (Figure 5). 

An SAE is defined by the EMA as any medical occurrence that at any dose, results in death, is life-threatening, requires inpatient hospitalization or prolongs an existing hospitalization, results in persistent or significant disability/incapacity, is a congenital anomaly/birth defect, or is an important medical event that may jeopardize the patient or require intervention to prevent the aforementioned outcomes [31]. No SAEs related to the study vaccines were reported. 

Overall, the frequency of solicited local and systemic AEs was higher in the BNT162b2 XBB.1.5 arm (32.8%) compared to the PHH-1V81 arm (27.9%) (Table S8).

## 4. Discussion

The interim analysis compared the immunogenicity of PHH-1V81 with BNT162b2 XBB.1.5 against Omicron XBB 1.16. Secondary endpoints and an additional analysis evaluated neutralization titers against XBB.1.5 and Omicron JN.1. The results indicated that 14 days post-vaccination, the immune response against XBB.1.16 with the PHH-1V81 booster was consistently not only non-inferior but also superior to that with the BNT162b2 XBB.1.5 booster. Additionally, PHH-1V81 significantly increased neutralizing antibody titers against XBB.1.5 and JN.1. GMT ratios, suggesting a superior antibody response with PHH-1V81 compared to BNT162b2 XBB.1.5 against XBB.1.5, while maintaining non-inferiority against JN.1. It is noteworthy that the JN.1 VNA analysis was performed with a smaller subset of participants analyzed, and the GMT ratio was close to superiority. Moreover, upon PHH-1V81 vaccination, the RBD-responding IFN-γ-producing T-cells showed specificity not only against the homologous Omicron XBB.1.16 vaccine variant but also cross-reactivity against the Omicron XBB.1.5, BA.1, BA.2.86, and JN.1 variants and the ancestral Wuhan strain.

Subgroup analyses of neutralizing antibody responses against Omicron XBB.1.16 and Omicron XBB.1.5 among individuals aged 60 years or older, both with and without prior reported SARS-CoV-2 infections and subjects who received three or more prior doses, showed higher antibody titers with PHH-1V81 compared to BNT162b2 XBB.1.5. Safety endpoint frequencies were similar between the two booster groups, but PHH-1V81 demonstrated an overall lower reactogenicity profile, with a significantly higher proportion of subjects reporting no AE compared to BNT162b2 XBB.1.5. This aligns with the previously reported favorable safety profile of PHH-1V compared to BNT162b2 [18]. It reinforces the notion that adjuvanted protein subunit vaccines, like PHH-1V81, are well-suited for vulnerable populations, including immunocompromised individuals, due to their safety profile and their ability to generate high levels of neutralizing antibodies, surpassing those induced by inactivated virus vaccines [32]. 

Other concerning, very rare AEs (<1 in 10,000), such as myocarditis reported with mRNA vaccine platforms, adjuvanted protein-based vaccines, and adenovirus vector-based vaccines, have not been observed in previous studies with the PHH-1V prototype vaccine [33,34]. According to currently available evidence, most cases of vaccine-associated myocarditis are mild, transient, and self-limiting. However, these infrequent AEs can have a worse prognosis in young males after two doses of mRNA vaccines, making them a more vulnerable population [35,36]. Innate immunity, cytokines, and the inflammatory reaction may all play a crucial role [33]. The PHH-1V and adapted PHH-1V81 vaccines have shown low reactogenicity, with no cases documented in previous studies [18,19,25,37]. Similarly, a low incidence of myocardial events has also been observed with other protein-based vaccines [33]. In this study, the adapted PHH-1V81 booster has been less reactogenic than the adapted BNT162b2 XBB.1.5 booster. The low reactogenicity of PHH-1V and the adapted PHH-1V81, coupled with the low incidence of myocardial events documented with protein-based vaccines, has not justified the measurement of troponin levels, other biomarkers, or other inflammatory parameters in this clinical trial. Pharmacovigilance registries will likely provide sufficient data to better characterize the epidemiology of these rare AEs and their association with COVID-19 immunization [33].

The immunogenicity of PHH-1V81 against Omicron’s XBB.1.16 and XBB.1.5 variants holds significant epidemiological importance amid Omicron’s emergence as the predominant SARS-CoV-2 variant globally [38]. The ongoing evolution of SARS-CoV-2 and the emergence of new variants, exemplified by the JN.1 variant, pose challenges with waning or inadequate protection from previous infections and vaccines, potentially allowing for the evasion of immunity and enhancing transmission [6,39]. This is compounded by a population largely exposed to multiple variants through infection, vaccination, and boosting [40,41]. Consequently, future vaccines may encounter challenges related to factors such as immune imprinting, immune seniority [42], or an immunoglobulin G (IgG) class switch [43].

In this complex scenario, it can be hypothesized that the squalene adjuvanted RBD-based vaccine can stimulate the innate immune system [44,45], and additionally, the RBD immune-focused approach can induce a better response against new and conserved epitopes in emerging variants [46,47], overcoming or minimizing the potential negative effects associated with previous exposures. Furthermore, the IgG4 class switch associated with repeated vaccination with mRNA vaccines suggests the potential for alternative platforms for subsequent immunizations [43,48]. The results support these assumptions, as better responses were observed with PHH-1V81 compared to BNT162b2 XBB.1.5 against XBB and JN.1. The data presented herein, 14 days post-booster, do not include the IgG subclass analysis, as it is planned to be conducted at trial termination. This will include longer time points and provide better insights of the IgG4 class switch, which is mostly reported at later time points [41,46].

These interim results align with earlier findings of broad immune responses against previously circulating variants of concern, including Wuhan-Hu-1, Beta, Delta, and Omicron BA.1 observed with PHH-1V (Bimervax^®^; HIPRA) [18,37]. They underscore the value of incorporating alternative vaccine platforms and adjuvants to address the diminishing returns observed with successive mRNA vaccinations, ensuring sustained immunogenicity against evolving variants of the virus [49,50]. In this interim analysis, established laboratory techniques, including PBNA, VNA, and ELISpot, were employed to evaluate immune responses. However, limitations stem from the interim nature of the results, the short follow-up period (14 days post-booster), limited statistical power for subgroup analyses, and a restricted representation of individuals aged 60 and older. Long-term observations of the effects of the PPH-1V81 booster will be conducted at trial termination, including assessments of IgG subclasses, particularly IgG4 levels, which peak around six months post-vaccination [43,48,50,51]. 

## 5. Conclusions

The interim results endorse PHH-1V81 as a promising booster, offering balanced immunogenicity and tolerability. This updated vaccine is key for addressing SARS-CoV-2 variability and population immunity, enhancing protection against current and emerging strains. Future research should prioritize a prolonged follow-up to confirm immune response persistence and effectiveness, especially among vulnerable groups. With a favorable safety profile and robust response against tested variants, including predominant JN.1, the adjuvanted RBD SARS-CoV-2 vaccine emerges as a compelling candidate for future COVID-19 vaccination strategies.

## Figures and Tables

**Table 1:** The baseline demographics of the safety population by vaccine arm.

	PHH-1V81 (N = 536)	BNT162b2 XBB.1.5 (N = 264)	Total (N = 800)
Age			
n	535	264	799
Mean (SD; range), years	44.7 (15.5; 18–88)	44.9 (15.0; 18–86)	44.7 (15.3; 18–88)
<60 years old, n (%)	463 (86.4%)	233 (88.3%)	696 (87.0%)
≥60 years old, n (%)	73 (13.6%)	31 (11.7%)	104 (13.0%)
Gender			
Female, n (%)	322 (60.1%)	152 (57.6%)	474 (59.3%)
Race			
White, n (%)	501 (93.5%)	249 (94.3%)	750 (93.8%)
Native Hawaiian or other Pacific Islander, n (%)	1 (0.2%)	0 (0.0%)	1 (0.1%)
No reported, n (%)	34 (6.3%)	15 (5.7%)	49 (6.1%)
Prior reported COVID-19 infection			
Yes	278 (51.9%)	134 (50.8%)	412 (51.5%)
No	258 (48.1%)	130 (49.2%)	388 (48.5%)
Previous vaccine doses, n (%)			
3 doses	358 (66.8%)	177 (67.0%)	535 (66.9%)
4 doses	177 (33.0%)	87 (33.0%)	264 (33.0%)
5 doses	1 (0.2%)	0 (0.0%)	1 (0.1%)

N: the number of subjects in the population; n: the number of subjects meeting the criterion, SD: Standard deviation.

## Data Availability

The datasets for this study are available upon reasonable request to the corresponding author with the permission of HIPRA S.A. Please contact Silvina Natalini Martínez at slnatalini@hmhospitales.com to request access to the data.

## Supplementary Materials

The following supporting information is available online at https://www.mdpi.com/article/10.3390/vaccines12080840/s1, Table S1. Inclusion and Exclusion Criteria. Table S2. An analysis of neutralizing antibodies against the Omicron XBB.1.16 and Omicron XBB.1.5 variants on day 14 post-vaccination boost in the mITT population. Table S3. Analysis of neutralizing antibodies against Omicron XBB.1.16 and Omicron XBB.1.5 variants in persons ≥60 years-old at baseline and day 14 (mITT population). Table S4. Analysis of neutralizing antibodies against Omicron XBB.1.16 and Omicron XBB.1.5 variants in persons with and without prior reported SARS-CoV-2 infections at baseline and day 14 (mITT population) by vaccine arm (mITT population). Table S5. Analysis of neutralizing antibodies against Omicron XBB.1.16 and Omicron XBB.1.5 variants in persons with 3 or ≥4 prior doses of a COVID-19 vaccine at baseline and day 14 by vaccine arm (mITT population). Table S6. Analysis of neutralizing antibodies against Omicron JN.1 variant by VNA. Table S7. Analysis of IFN-γ-producing T cells upon PBMC re-stimulation with SARS-CoV-2-derived peptide pools by ELISpot. Table S8. Solicited systemic and local adverse events from day 0 through day 7 of the safety population that completed day 14 visit by vaccine arm and type of adverse event.
