# Supplementary material for: Omicron XBB.1.16-Adapted Vaccine for COVID-19: Interim Immunogenicity and Safety Clinical Trial Results
Source: Vaccines (Basel). 2024 Jul 25;12(8):840. doi: 10.3390/vaccines12080840 (PMC11359014; doi:10.3390/vaccines12080840)
Supplement: Supplementary file 1 [file vaccines-12-00840-s001.zip › vaccines-3082976-supplementary.pdf]

Supplementary Material

Omicron XBB.1.16-Adapted Vaccine for COVID-19: Interim Immunogenicity and Safety Clinical Trial Results

Content

Table S1. Inclusion and Exclusion Criteria....2

Table S2. Analysis of neutralizing antibodies against Omicron XBB.1.16 and Omicron XBB.1.5 variants on Day 14 post-vaccination boost in the mITT population 4

Table S3. Analysis of neutralizing antibodies against Omicron XBB.1.16 and Omicron XBB.1.5 variants in persons ≥60 years-old at Baseline and day 14 (mITT population)....5

Table S4. Analysis of neutralizing antibodies against Omicron XBB.1.16 and Omicron XBB.1.5 variants in persons with and without prior reported SARS-CoV-2 infections at baseline and day 14 (mITT population), by vaccine arm (mITT population) 6

Table S5. Analysis of neutralizing antibodies against Omicron XBB.1.16 and Omicron XBB.1.5 variants in persons with 3 or ≥4 prior doses of a COVID-19 vaccine at baseline and day 14, by vaccine arm (mITT population) 7

Table S6. Analysis of neutralizing antibodies against Omicron JN.1 variant by VNA 8

Table S7. Analysis of IFN-γ producing T cells upon PBMC re-stimulation with SARS-CoV-2 derived peptide pools by ELISpot....9

Table S8. Solicited systemic and local adverse events from day 0 through day 7 of the safety population that completed day 14 visit, by vaccine arm and type of adverse event....10

Table S1. Inclusion and Exclusion Criteria

Inclusion Criteria	Exclusion Criteria
1. Participants must have met all the following criteria to be considered eligible for the study:<br>2. Adults aged 18 or older at Day 0.<br>3. Were willing and able to sign the informed consent and could comply with all study visits and procedures.<br>4. Participants must had received a primary scheme of an EU-approved mRNA vaccine (2 doses) and at least one booster dose with an EU-approved mRNA vaccine. Last booster dose must had been administered at least 6 months before Day 0.<br>5. Had a negative Rapid Antigen Test for COVID-19 at Day 0 prior to vaccination.<br>6. Adults determined by clinical assessment, including medical history and clinical judgement, to be eligible for the study, including adults with pre-existing chronic and stable diseases (non-immunocompromised), if these were stable and well-controlled according to the investigator's judgement.<br>7. Participants biologically able to have children may have been enrolled in the study if the participant fulfilled all the following criteria:<br>• Had a negative urine pregnancy test at Day 0, only for those participants who were biologically able to become pregnant.<br>• Had practiced adequate contraception or had abstained from all activities that could result in pregnancy for at least 28 days prior to the study treatment, only for those participants who were biologically able to become pregnant.<br>• Had agreed to continue adequate contraception or abstinence through 3 months following the booster dose.<br>*- Participants with female reproductive system:*<br>i. Hormonal contraception [progesterone-only or combined: oral,<br>ii. injectable or transdermal (patch)]<br>iii. Intrauterine device	Participants who met any of the following criteria were excluded from participation in this study:<br>1. Acute illness with fever ≥ 38.0ºC at Day 0 or within 24 hours prior to vaccination. Afebrile participants with minor illnesses could be enrolled at the discretion of the investigator.<br>2. Other medical or psychiatric condition including recent (within the past year) or active suicidal ideation/behaviour that may have increased the risk of study participation or, in the investigator's judgement, made the participant inappropriate for the study.<br>NOTE: This includes both conditions that may increase the risk associated with study intervention administration or a condition that may interfere with the interpretation of study results.<br>3. History of severe adverse reaction associated with a vaccine and/or severe allergic reaction (e.g., anaphylaxis) to any component of the study intervention.<br>4. Immunocompromised individuals defined as those with primary and secondary immune deficiencies and those receiving chemotherapy or immunosuppressant drugs other than steroids and glucocorticoids (maximum 30mg/day of prednisone, or equivalent, by any administration route for a maximum of 30 consecutive days), within 90 days prior to vaccination.<br>5. Bleeding diathesis or condition associated with prolonged bleeding that would, in the opinion of the investigator, contraindicate intramuscular injection.<br>6. Receipt of blood-derived immune globulins, blood, or blood-derived products in the past 3 months.<br>7. Participation in other studies involving study intervention if last dose was within 28 days prior to screening and/or it was planned to receive during study participation.<br>8. Received any non-study vaccine within 14 days before or after screening. For live or attenuated vaccines, 4 weeks before or after screening.<br>9. Received any COVID-19 vaccines other than EU-approved mRNA vaccines.<br>10. Received any Omicron XBB adapted vaccine before Day 0.

Inclusion Criteria	Exclusion Criteria
iv. Vasectomized partner (the vasectomized partner should be the sole<br>v. partner for that participant).<br>vi. Condom.<br>*- Participants with male reproductive system:*<br>i. Vasectomized participants.<br>ii. Agreed to use condom in partners biologically able to become<br>iii. pregnant.	11. COVID-19 infection diagnosed in the previous 6 months before Day 0. History of COVID-19 infections was allowed.<br>History of a diagnosis or other conditions that, in the judgement of the investigator, may have affected study endpoint assessment or compromise participant safety.

Table S2. Analysis of neutralizing antibodies against Omicron XBB.1.16 and Omicron XBB.1.5 variants on Day 14 post-vaccination boost in the mITT population

	**Omicron XBB.1.16**			**Omicron XBB.1.5**		
	**GMT**		**GMFR**	**GMT**		**GMFR**
	**Baseline**	**Day 14**	**Day 14**	**Baseline**	**Day 14**	**Day 14**
**PHH-1V81** (N=406)	152.46 (134.72, 172.54)	1946.38 (1708.44, 2217.46)	12.76 (11.01, 14.78)	151.93 (134.89, 171.13)	1888.89 (1676.99, 2127.57)	12.42 (10.62, 14.51)
**BNT62b2 XBB.1.5** (N=193)	161.57 (136.40, 191.37)	1512.21 (1261.72, 1812.44)	9.42 (7.61, 11.66)	167.89 (142.04, 198.44)	1486.03 (1257.25, 1756.45)	8.88 (7.20, 10.94)
**Ratio** (95% CI) **BNT vs PHH**	1.06 (0,87 - 1,29)	0.78 (0.63, 0.96)	0.74 (0.57, 0.96)	1.1 (0.90, 1.35)	0,79 (0.64, 0.96)	0.71 (0.56, 0.92)
**p-value** for ratio = 1	0.570	0.0213	0.0218	0.330	0.0195	0.0082

N refers to subjects with data; GMT is shown as adjusted treatment mean (95% CI); GMT ratio is shown as BNT162b2 XBB vs PHH-1V81 (95% CI); GMFR is shown as fold rise of adjusted treatment means between timepoints (95% CI); GMFR ratio is shown as BNT162b2 XBB.1.5 vs PHH-1V81 [95% CI].

CI: confidence interval; GMT: Geometric Mean Titre; GMFR: Geometric Mean Fold Rise.

Table S3. Analysis of neutralizing antibodies against Omicron XBB.1.16 and Omicron XBB.1.5 variants in persons ≥60 years-old at Baseline and day 14 (mITT population)

Variable	PHH-1V81 (N=52)	BNT162b2 XBB.1.5 (N=23)
**Omicron XBB.1.16**		
GMT (95% CI) at baseline	127.94 (77.24; 211.91)	174.81 (91.99; 332.19)
GMT (95% CI) at day 14	1979.2 (1194.91; 3278.25)	1822.30 (958.92; 3463.01)
**Omicron XBB.1.5**		
GMT (95% CI) at baseline	136.61 (84.81; 220.05)	173.46 (95.89; 313.80)
GMT (95% CI) at day 14	1817.99 (1128.63; 2928.39)	1692.08 (935.35, 3061.04)
GMT is shown as adjusted treatment mean (95% CI)<br>CI: confidence interval; GMT: Geometric Mean Titre		

Table S4. Analysis of neutralizing antibodies against Omicron XBB.1.16 and Omicron XBB.1.5 variants in persons with and without prior reported SARS-CoV-2 infections at baseline and day 14 (mITT population), by vaccine arm (mITT population)

Variable	With prior reported SARS-CoV-2 infection		Without prior reported SARS-CoV-2 infection	
	PHH-1V81 (N=206)	BNT162b2 XBB.1.5 (N=99)	PHH-1V81 (N=200)	BNT162b2 XBB.1.5 (N=94)
**Omicron XBB.1.16**				
GMT (95% CI) at baseline	146.24 (123.06; 173.79)	161.17 (127.18; 204.24)	158.90 (130.95; 192.82)	164.18 (126.1; 213.83)
GMT (95% CI) at day 14	1871.73 (1575.01; 2224.37)	1493.63 (1178.65; 1892.78)	2026.55 (1670.08; 2459.10)	1562.60 (1199.81; 2035.07)
**Omicron XBB.1.5**				
GMT (95% CI) at baseline	148.38 (125.50; 175.43)	166.05 (131.99; 208.91)	154.17 (126.32; 188.15)	171.63 (131.88; 223.35)
GMT (95% CI) at day 14	1880.24 (1590.31; 2223.01)	1434.93 (1140.60; 1805.21)	1879.39 (1539.94; 2293.68)	1558.09 (1197.28; 2027.63)

GMT is shown as adjusted treatment mean (95% CI)
CI: confidence interval; GMT: Geometric Mean Titre; SARS-CoV-2: severe acute respiratory syndrome coronavirus 2

Table S5. Analysis of neutralizing antibodies against Omicron XBB.1.16 and Omicron XBB.1.5 variants in persons with 3 or ≥4 prior doses of a COVID-19 vaccine at baseline and day 14, by vaccine arm (mITT population)

Variable	3 prior doses of a COVID-19 vaccine		≥4 prior doses of a COVID-19 vaccine	
	**PHH-1V81 (N=272)**	**BNT162b2 XBB.1.5 (N=129)**	**PHH-1V81 (N=134)**	**BNT162b2 XBB.1.5 (N=64)**
**Omicron XBB.1.16**				
GMT (95% CI) at baseline	146.77 (125.99; 170.98)	168.96 (136.29; 209.47)	162.77 (127.31; 208.11)	145.07 (105.42; 199.63)
GMT (95% CI) at day 14	2115.66 (1816.08; 2464.66)	1504.10 (1213.26; 1864.66)	1628.06 (1273.39; 2081.50)	1516.10 (1101.72; 2086.33)
**Omicron XBB.1.5**				
GMT (95% CI) at baseline	143.93 (124. 31; 166.65)	171.71 (139.57; 211.26)	165.80 (129.49; 212.28)	157.51 (114.92; 215.87)
GMT (95% CI) at day 14	2081.44 (1797.67; 2409.99)	1524.36 (1238.99; 1875.45)	1516.64 (1184.53; 1941.87)	1385.97 (1011.19; 1899.64)

GMT is shown as adjusted treatment mean (95% CI)
CI: confidence interval; COVID-19: Coronavirus disease 2019; GMT: Geometric Mean Titre

Table S6. Analysis of neutralizing antibodies against Omicron JN.1 variant by VNA

	Omicron JN.1 by VNA		
	GMT		GMFR
	Baseline	Day 14	Day 14
**PHH-1V81**<br>(N=65)	58.51<br>(43.32, 79.02)	768.44<br>(568.96, 1037.86)	13.34<br>(8.84, 20.12)
**BNT162b2 XBB.1.5**<br>(N=35)	53.02<br>(36.13, 77.82)	505.88<br>(344.70, 742.43)	9.27<br>(5.70, 15.07)
**Ratio** (95% CI)<br>**BNT vs PHH**	0.91<br>(0.59, 1.39)	0.66<br>(0.43, 1.01)	0.69<br>(0.42, 1.14)
**p-value** for ratio = 1	0.6486	0.0540	0.1474

N refers to subjects with data; GMT is shown as adjusted treatment mean (95% CI); GMT ratio is shown as BNT162b2 XBB vs PHH-1V81 (95% CI) followed by p-value for ratio = 1 ; GMFR is shown as fold rise of adjusted treatment means between timepoints (95% CI); GMFR ratio is shown as BNT162b2 XBB.1.5 vs PHH-1V81 [95% CI] followed by p-value for ratio = 1.

CI: confidence interval; GMT: Geometric Mean Titre; GMFR: Geometric Mean Fold Rise.

Table S7. Analysis of IFN-γ producing T cells upon PBMC re-stimulation with SARS-CoV-2 derived peptide pools by ELISpot

	PHH-1V81	BNT162b2 XBB.1.5
**RBD Omicron XBB.1.16**		
n	27	12
Median (Q1; Q3) at baseline	33.75 (14.38; 64.38)	31.25 (21.56; 57.19)
Median (Q1; Q3) at day 14	76.25 (24.38; 118.75)	56.88 (163.44; 35.00)
Difference from baseline (SD)	0.0029 (0.00093)	0.0029 (0.00140)
p-value for difference = 0	0.0037	0.0454
**RBD Omicron XBB.1.5**		
n	27	12
Median (Q1; Q3) at baseline	43.75 (16.25; 61.88)	22.50 (14.06; 50.94)
Median (Q1; Q3) at day 14	60.00 (25.63; 128.13)	53.13 (35.00; 133.13)
Difference from baseline (SD)	0.0024 (0.00092)	0.0033 (0.00137)
p-value for difference = 0	0.0139	0.0229
**RBD Omicron JN.1**		
n	26	13
Median (Q1; Q3) at baseline	18.13 (7.71; 39.38)	22.50 (8.75; 65.00)
Median (Q1; Q3) at day 14	36.88 (16.56; 66.56)	32.50 (17.50; 61.25)
Difference from baseline (SD)	0.0019 (0.00051)	0.0015 (0.00072)
p-value for difference = 0	0.0007	0.0388
Q1: lower quartile; Q3: upper quartile; SD: Standard deviation		

Table S8. Solicited systemic and local adverse events from day 0 through day 7 of the safety population that completed day 14 visit, by vaccine arm and type of adverse event

Type of events	PHH-1V81 (N=409)		BNT162b2 XBB.1.5 (N=198)		Overall (N=607)	
	**Events**	**Subjects (%)**	**Events**	**Subjects (%)**	**Events**	**Subjects (%)**
**Overall systemic events**	**282**	**114 (27.9)**	**155**	**65 (32.8)**	**437**	**179 (29.5)**
Headache	80	71 (17.4)	45	40 (20.2)	125	111 (18.3)
Fatigue	60	56 (13.7)	35	32 (16.2)	95	88 (14.5)
Muscle Pain	54	48 (11.7)	25	24 (12.1)	79	72 (11.9)
Malaise/Discomfort	36	33 (8.1)	25	24 (12.1)	61	57 (9.4)
Diarrhoea	16	15 (3.7)	6	6 (3.0)	22	21 (3.5)
Nausea/ Vomiting	14	13 (3.2)	5	5 (2.5)	19	18 (3.0)
Enlarged lymphnodes (lymphadenopathy)	11	11 (2.7)	6	6 (3.0)	17	17 (2.8)
Axillary pain	9	9 (2.2)	7	7 (3.5)	16	16 (2.6)
Fever	2	2 (0.5)	1	1 (0.5)	3	3 (0.5)
**Overall local events**	**502**	**214 (52.3)**	**284**	**120 (60.6)**	**786**	**334 (55.0)**
Injection site pain / tenderness/ discomfort	362	210 (51.3)	201	117 (59.1)	563	327 (53.9)
Injection site induration / swelling	85	65 (15.9)	58	42 (21.2)	143	107 (17.6)
Injection site erythema / redness	55	46 (11.2)	25	22 (11.1)	80	68 (11.2)
